# Identification of neisserial DNA binding components

**DOI:** 10.1099/mic.0.022640-0

**Published:** 2009-03

**Authors:** Emma Lång, Kristine Haugen, Burkhard Fleckenstein, Håvard Homberset, Stephan A. Frye, Ole Herman Ambur, Tone Tønjum

**Affiliations:** 1 Centre for Molecular Biology and Neuroscience, Institute of Microbiology, University of Oslo, N-0027 Oslo, Norway; 2 Centre for Molecular Biology and Neuroscience, Institute of Microbiology, Rikshospitalet, Oslo, Norway; 3 Institute of Immunology, University of Oslo, N-0027 Oslo, Norway

## Abstract

*Neisseria meningitidis*, a causative agent of meningitis and septicaemia, expresses type IV pili, a feature correlating with the uptake of exogenous DNA from the environment by natural transformation. The outer membrane complex PilQ, through which pili are extruded and retracted, has previously been shown to bind DNA in its pore region. In order to further elucidate how DNA is transported across the membranes, we searched for DNA binding proteins within the meningococcal inner membrane. Inner membrane fractions from a panel of neisserial strains were subjected to a solid-phase overlay assay with DNA substrates, and MS was subsequently employed to identify proteins that bind DNA. A number of DNA binding components were detected, including the pilus biogenesis component PilG, the competence protein ComL, and the cell division ATP-binding protein FtsE, as well as two hypothetical proteins. The DNA binding activity of these components was not dependent on the presence of the neisserial DNA uptake sequence. Null mutants, corresponding to each of the proteins identified, were constructed to assess their phenotypes. Only mutants defective in pilus biogenesis were non-competent and non-piliated. The DNA binding activity of the pilus biogenesis components PilQ and PilG and the phenotypes of their respective null mutants suggest that these proteins are directly involved as players in natural transformation, and not only indirectly, through pilus biogenesis.

## INTRODUCTION

*Neisseria meningitidis*, the meningococcus, is one of the leading causative agents of meningitis and septicaemia worldwide (Davidsen *et al.*, 2007a). Horizontal gene transfer mediates the exchange of genetic information between bacterial strains and is driven by three processes: conjugation, transformation and transduction. Among these, transformation, which is the uptake and incorporation of exogenous DNA into the bacterial chromosome, is the main source of new DNA introduced into neisserial genomes (Koomey, 1998). The transformation process is dependent on type IV pilus expression (Swanson *et al.*, 1971), the presence of the neisserial DNA uptake sequence (DUS) (5′ ATGCCGTCTGAA 3′) in the incoming DNA (Ambur *et al.*, 2007; Goodman & Scocca, 1988; Mathis & Scocca, 1984) and RecA-dependent homologous recombination (Koomey & Falkow, 1987).

Unlike other Gram-negative bacteria, *N. meningitidis* is naturally competent for transformation throughout its entire life cycle (Jyssum & Lie, 1965), provided that it expresses type IV pili, which are filamentous, hair-like appendages emanating from the bacterial surface (Frøholm *et al.*, 1973). This association has also been observed in other bacteria, such as *Neisseria gonorrhoeae* (Biswas *et al.*, 1977; Mathis & Scocca, 1984; Swanson *et al.*, 1971), *Eikenella corrodens* (Tønjum *et al.*, 1993), *Legionella pneumophila* (Stone & Kwaik, 1999), *Pseudomonas stutzeri* (Lorenz & Wackernagel, 1990), *Thermus thermophilus* (Friedrich *et al.*, 2002) and *Moraxella nonliquefaciens* (Bøvre, 1964; Bøvre *et al.*, 1970). Type IV pilus expression also has a role in adherence (Swanson *et al.*, 1971; Swanson, 1973), twitching motility (Henrichsen *et al.*, 1972; Mattick, 2002), biofilm formation (O'Toole & Kolter, 1998), bacteriophage infection (Bradley, 1974) and virulence (Bieber *et al.*, 1998; Comolli *et al.*, 1999; Merz *et al.*, 1999; Pujol *et al.*, 1999). Twitching motility is caused by type IV pilus retraction, which is dependent on the ATPase PilT (Merz *et al.*, 2000; Wolfgang *et al.*, 1998).

The biogenesis of type IV pili is dependent on a complex machinery of proteins. A number of proteins required for neisserial transformation have been described, including the secretin PilQ, through which pili are extruded and retracted (Collins *et al.*, 2005; Frye *et al.*, 2006), as well as the competence factors ComA (Facius & Meyer, 1993), ComE (Chen & Gotschlich, 2001) and ComL (Fussenegger *et al.*, 1996). In addition to PilQ, the inner membrane proteins PilG and PilP have previously been found to be essential for pilus biogenesis and, thus, for competence (Balasingham *et al.*, 2007; Tønjum *et al.*, 1995). However, PilQ is the only pilus biogenesis component that has previously been shown to bind DNA (Assalkhou *et al.*, 2007).

The uptake of DNA into the meningococcal cell can be dissected into several steps that encompass crossing of the outer and inner membranes and genome incorporation. Although the neisserial transformation pathway has been described to some extent, little is known about how the transforming DNA is taken up. It is still a conundrum whether the effect of pilus biogenesis components on transformation is of a direct or only of an indirect nature. We suggest that meningococcal transformation is coupled to pilus retraction and that exogenous DNA is taken up through non-specific attachment to retracting pili, while other DNA binding components, such as the outer membrane protein PilQ, promote further entry of DNA into the meningococcal cell.

Thus, pili, pilus biogenesis components and DNA binding proteins act together in the uptake of exogenous DNA into the meningococcal cell, enforced by pilus retraction. In order to pursue this hypothesis, we searched for DNA binding proteins that co-purify with the *N. meningitidis* inner membrane fraction. DNA binding proteins were detected by solid-phase overlay assays using DNA substrates with or without a DUS. Subsequently, the proteins that bound DNA were identified by MS. The corresponding null mutants were tested to see if they had defects in transformation. The identification of novel DNA binding components might contribute to further elucidation of the transformation process, and of other aspects of DNA metabolism.

## METHODS

### Bacterial strains and growth conditions.

Bacterial strains employed in the study are listed in Table 1. All neisserial strains were grown on blood agar or GC plates at 37 °C in 5 % CO_2_, while *Escherichia coli* strains were cultivated on LB plates at 37 °C.

Selective antibiotics were added when required.

### Cellular fractionation and total membrane enrichment.

Neisserial membrane fractionation was performed according to the method of Pannekoek *et al.* (1992) with some modifications as follows. Meningococcal cells were resuspended in 180 ml phosphate-buffered saline (PBS), pH 7.5, and collected by centrifugation at 4000 ***g*** for 20 min at 4 °C. The cells were resuspended in 30 ml 50 mM Tris/HCl, pH 8.0, and subjected to one round of freeze–thawing at −20 °C and room temperature. Additional cell lysis was conducted by passing the suspension twice through a French press (103 500 kPa), Thermo Electron. Undisrupted cells were removed by centrifugation at 4000 ***g*** for 25 min at 4 °C. Remaining cell fragments were discarded by centrifugation twice at 10 000 ***g*** for 15 min at 4 °C. The membrane fraction was collected by ultracentrifugation at 215 000 ***g*** for 2 h at 4 °C. The membrane pellet was resuspended in 13 ml 50 mM Tris/HCl, pH 8.0. After an additional hour of ultracentrifugation at 215 000 ***g*** and 4 °C, the final pellet was resuspended in ∼200 μl distilled water.

### Inner membrane isolation.

For solubilization and separation of inner and outer membranes from the membrane fraction, we employed a method based on the ability of *N*-lauroylsarcosine (Sarkosyl) to selectively solubilize inner membrane proteins (Brossay *et al.*, 1994; Filip *et al.*, 1973; Mietzner *et al.*, 1984). Total membrane fractions (corresponding to 500 μg protein) were washed in 4 ml HEPES buffer, pH 7.4. After 1 h ultracentrifugation at 100 000 ***g*** and 4 °C, the pellet was resuspended in 250 μl 10 mM HEPES buffer using a 25 gauge needle. Two-hundred and fifty microlitres of 0.4 % Sarkosyl in 10 mM HEPES buffer was added, giving a final concentration of 0.2 % Sarkosyl, equivalent to 1 μg detergent (μl protein)^−1^. Incubation at room temperature for 10 min was followed by 1 h ultracentrifugation at 100 000 ***g*** and 4 °C. The supernatant, consisting of solubilized inner membrane proteins, was collected, and the pellet was incubated in 0.2 % Sarkosyl and centrifuged once more to remove residual inner membrane proteins. The remaining pellet, constituting the outer membrane fraction, was resuspended in 500 μl 10 mM HEPES buffer, and after 1 h ultracentrifugation at 100 000 ***g*** and 4 °C, the final pellet was resuspended in ∼200 μl 10 mM HEPES buffer. Both inner membrane and outer membrane fractions were stored at −70 °C.

### Isolation of outer membrane vesicles (OMVs).

Isolation of OMVs from *N. meningitidis* has previously been described (Balasingham *et al.*, 2007; Frasch & Mocca, 1978). In short, bacteria were grown on blood agar plates, harvested in serotype antigen buffer (0.2 M LiCl, 0.1 M sodium acetate, pH 5.8) and inactivated at 60 °C for 30 min. A bacterial suspension of OD_600_ 20 was prepared and shaken with 2 mm glass beads at 230 r.p.m. for 15 min at room temperature. Cellular debris was removed by two rounds of centrifugation at 4000 ***g*** for 20 min and 18 000 ***g*** for 15 min, respectively. The supernatant was subjected to ultracentrifugation at 140 000 ***g*** for 90 min, the pellet was resuspended in distilled water, and another round of ultracentrifugation at 140 000 ***g*** for 90 min was performed. The resulting pellet was resuspended in distilled water and stored at −70 °C.

### Determination of protein concentration in membrane fractions.

The protein content in neisserial membrane fractions was determined using a detergent-compatible (DC) protein assay (Bio-Rad Laboratories), which is a modification of the Lowry assay.

### Immunoblotting of meningococcal membrane fractions and purified proteins.

A 1 μg sample of purified meningococcal inner and outer membrane proteins, as well as OMV proteins and recombinant PilG protein (E. Lång and others, unpublished results), were separated by SDS-PAGE and transferred onto nitrocellulose membranes (Hybond-C Extra, Amersham GE Healthcare) in Towbin transfer buffer (25 mM Tris/HCl, 192 mM glycine, 20 % methanol, 0.1 % SDS, pH 8.3). Rabbit polyclonal antibodies directed against PilG (E. Lång and others, unpublished results) and PilQ (Tønjum *et al.*, 1998) were used in immunoblotting at dilutions of 1 : 1000 and 1 : 2500, respectively. Procedures for SDS-PAGE and immunoblotting have been previously described (Collins *et al.*, 2005; Tønjum *et al.*, 1998).

### Solid-phase overlay assay for protein–DNA interaction.

Protein–DNA interactions were assessed by a solid-phase overlay assay (South-western analysis) (Assalkhou *et al.*, 2007). In short, 3–5 μg of isolated inner membrane proteins and 0.2 μg recombinant PilG protein were separated by SDS-PAGE and transferred to membranes as described above. The membranes were pre-incubated in renaturation buffer (0.25 % gelatin, 0.5 % BSA, 0.2 % Triton X-100, 10 mM Tris/HCl, 5 mM *β*-mercaptoethanol, 100 mM NaCl, pH 7.5) at room temperature for 1 h prior to the addition of oligonucleotides. Approximately 400 pmol biotinylated DNA substrate (Table 2) was applied to each membrane, and incubation in renaturation buffer was conducted with shaking at 4 °C overnight. The next day, the membranes were incubated for an extra 2 h in renaturation buffer with oligonucleotides at room temperature. The membranes were washed in washing buffer (10 mM Tris/HCl, 100 mM NaCl, pH 7.5). Incubation with alkaline phosphatase (AP)-conjugated streptavidin (1 : 5000) was performed with shaking for 1 h at room temperature. Additional washing with final washing buffer (100 mM Tris/HCl, 0.9 % NaCl, pH 7.5) was then performed. Biotinylated DNA was detected using 5-bromo-4-chloro-3′-indolyl phosphate *p*-toluidine salt (BCIP) and nitro-blue tetrazolium chloride (NBT) as substrates for the AP. The DNA glycosylase Fpg was used as positive control for the detection of DNA binding and BSA as a negative control. Proteins migrating corresponding to DNA binding bands were excised from a Coomassie blue-stained SDS-PAGE gel, run in parallel with those used in South-western analysis. The excised proteins were pre-treated for MS analysis. Due to the close migration of some proteins in the inner membrane fractions, it was sometimes complicated to excise a single protein. Therefore, proteins were selectively excised from the gel based on the presence of distinct bands both on the Coomassie gel and on the South-western blot(s). The South-western experiments, with parallel SDS-PAGE, and subsequent MS analysis were repeated at least three times.

### Protein identification by peptide mass fingerprinting MALDI-TOF MS.

Proteins, in sections excised from the SDS-PAGE, were subjected to in-gel digestion with trypsin as previously described (Fleckenstein *et al.*, 2004). Tryptic peptide mixtures were desalted and concentrated by running the peptides through a GELoader tip (Eppendorf) column filled with minute amounts of Poros 20 R2 reversed-phase packing sorbent (Applied Biosystems). Finally, a solution containing 70 % acetonitrile (ACN), 0.1 % trifluoroacetic acid (TFA) and 10 mg *α*-cyano-4-hydroxycinnamic acid ml^−1^ was used to elute the peptides directly onto a stainless steel target plate (Bruker Daltonics). The samples were allowed to crystallize on the plate and analysed on an Ultraflex II MALDI-TOF/TOF mass spectrometer (Bruker Daltonics) operated in the positive reflector mode. MS lists obtained were searched by MASCOT (Matrix Science) against the NCBI, MSDB and Swiss-Prot databases with *N. meningitidis* as the selected taxon.

### Bioinformatics analyses and screening for DNA binding motifs.

Proteins identified by MS analysis were characterized by using different protein databases and bioinformatics tools. The PROSITE database (Hulo *et al.*, 2008) was used to screen for protein domains and functional sites, whereas EMBOSS (Rice *et al.*, 2000) was used to predict helix–turn–helix structures and define the charge predictions of the deduced amino acids of the proteins, by using the EMBOSS helix–turn–helix and charge package. Uniprot (Boeckmann *et al.*, 2003) provided information about properties of the proteins identified, and MicrobesOnline (Alm *et al.*, 2005) was used to study gene sequence, gene homologues and domain structures.

### *N. meningitidis* mutant construction.

Null mutants corresponding to each of the DNA binding proteins identified were constructed. DNA with homology 400–600 bp upstream and downstream of the target gene was PCR-amplified and ligated with a kanamycin-resistance gene cassette between the two PCR fragments (Menard *et al.*, 1993) into the plasmid pBluescript II SK(+) (pBSK+) through four-point ligation into relevant restriction sites, and plasmid DNA was propagated in *E. coli* ER2566 or XL-1 Blue (Table 1). The oligonucleotide primers employed are listed in Table 3. *N. meningitidis* strain M400 was transformed with plasmid DNA carrying the cloned DNA fragments, which recombined and integrated into the host chromosome, allowing each gene to be interrupted by the kanamycin-resistance gene. *N. meningitidis* transformants were selected by growth on plates containing 100 μg kanamycin ml^−1^ (Sigma), and the gene disruption(s) were confirmed by PCR and DNA sequence analysis.

### Phenotypic analysis of *N. meningitidis* null mutants.

*N. meningitidis* null mutants were compared with wild-type strains in phenotypic analyses.

**(i) Colony morphology.** *N. meningitidis* strains cultured on clear GC plates were assessed by stereo microscopy to define whether they had an agglutinating (agg+) or non-agglutinating (agg−) colonial morphology (Blake *et al.*, 1989).

**(ii) Purification of type IV pilus fibres.** Type IV pili were purified from the meningococcal cell surface using ammonium sulfate precipitation of a shearing fraction (Brinton *et al.*, 1978). Meningococcal cells from half a heavily streaked GC plate were resuspended in 1 ml 0.15 M ethanolamine buffer, pH 10.5, and vortexed for 1 min. Cellular debris was removed by centrifugation at 16 000 ***g*** for 30 min twice. Pili were precipitated from the supernatant by adding one-tenth of the total volume of saturated ammonium sulfate in 0.15 M ethanolamine buffer and left at room temperature overnight. Precipitated pili were collected by centrifugation for 15 min at 16 000 ***g***, and the pellet was washed twice in 1 ml 50 mM Tris/HCl, 150 mM NaCl, pH 8.0, before being dissolved in distilled water. Total protein was determined in the residual cells with a Bio-Rad DC protein assay. The pilus preparations were analysed on SDS-PAGE gels stained with Coomassie blue, using the total protein concentration from the residual cells to normalize the loading. Each null mutant was analysed in triplicate and the experiment was repeated at least three times.

**(iii) Competence screening.** Competence for transformation of wild-type and mutant strains was performed using the plasmid p1080 *mutY-erm^r^* as donor DNA (Table 1) (Davidsen *et al.*, 2007b). Wild-type and mutant strains were harvested in CO_2_-saturated liquid GC medium containing 7 mM MgCl_2_ and 1× IsoVitalex (Becton Dickinson Diagnostic Systems). The bacteria were exposed to either plasmid p1080 *mutY-erm^r^* or distilled water (negative control). Addition of 0.1 mg DNase I ml^−1^ (Sigma) mediated degradation of extracellular DNA before 10 volumes of liquid GC medium were added. The bacterial solutions were incubated with tumbling at 37 °C for 4.5 h and subsequently plated on both plain GC medium and GC medium containing 300 μg erythromycin (Erm) ml^−1^. The transformation rate was estimated by dividing the number of Erm-resistant c.f.u. by the total number of c.f.u. The assay was repeated at least three times for each null mutant.

## RESULTS

### Cellular fractionation and inner membrane isolation

**Fig. 1. f1:**
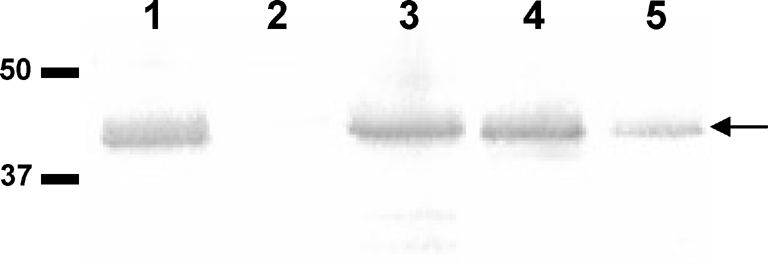
Identification of PilG in the inner membrane (IM). Immunoblotting of meningococcal membrane fractions localizes the PilG protein in the IM of *N. meningitidis*. Lanes: 1, H44/76 wild-type (wt) IM; 2, H44/76 ΔPilG IM; 3, Z2491 wt IM; 4, M1080 wt IM; 5, H44/76 wt outer membrane. The positions of the 37 and 50 kDa size standards are shown on the left. The arrow on the right indicates the position of the PilG protein.

Inner membrane fractions from a panel of *N. meningitidis* strains from three important serogroups (A, B and C) as well as *N. gonorrhoeae* strain N400 were enriched, using antibody-mediated PilG detection as a marker for inner membrane protein content (Fig. 1). Although Sarkosyl preferentially solubilizes inner membranes, complete separation of neisserial inner and outer membrane proteins is a challenging, if not impossible, task (Masson & Holbein, 1983). In addition to the inner membrane proteins enriched, some outer membrane proteins were also present in the neisserial inner membrane fractions, including PilQ, H.8 and Omp85. These proteins are normally expressed at high levels. The presence of outer membrane proteins within the inner membrane fraction was quantified by using PilQ as a marker (data not shown). In addition, some PilG protein could be detected in the outer membrane fraction (Fig. 1, lane 5), once again indicating that the separation of inner and outer membrane proteins was incomplete, due to the membrane composition and the limitations of the separation methods available. It must however be noted that the vast majority of PilG and other inner membrane proteins were found in the inner membrane fraction and vice versa for PilQ in the outer membrane fraction. The aim of this part of the study was to enrich inner membrane proteins into one fraction in our search for DNA binding proteins. For this purpose, using the detergent Sarkosyl was clearly more efficient than using Triton X-100 for neisserial inner membrane enrichment (data not shown).

### Detecting DNA binding proteins by a solid-phase overlay assay

**Fig. 2. f2:**
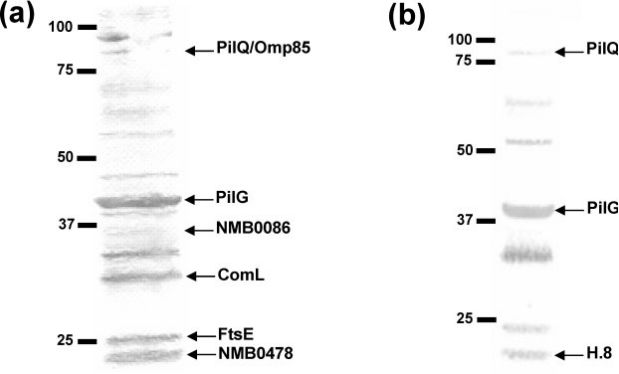
Detection of DNA binding components. Solid-phase overlay assay (South-western analysis) of neisserial inner membrane fractions with biotin-labelled DNA substrates was performed. (a) Identification of PilQ, Omp85, PilG, NMB0086, ComL, FtsE and NMB0478 in the inner membrane fraction of *N. meningitidis* strain H44/76. (b) Identification of PilQ, Omp85, PilG and H.8 in the inner membrane fraction of *N. meningitidis* strain 8013. The DNA substrates depicted were ssDNA substrate T_1_ (a) and T_3_ (b) with and without the 10 bp DUS, respectively. The positions of the size standards (kDa) are shown on the left.

Inner membrane fractions, representing lower complexity than using the entire cellular lysate as a target, from a panel of neisserial strains were searched for DNA binding proteins by solid-phase overlay assay in the form of a South-western analysis. All inner membrane fractions were tested with all the DNA substrates with or without a DUS listed in Table 2. Inner membrane fractions from all neisserial strains included in the study exhibited multiple DNA binding proteins. A selection of the proteins identified by MS is shown in Fig. 2. However, no apparent DUS specificity or preference for single-stranded or double-stranded DNA was detected among the candidate proteins identified. Protein bands to be characterized by subsequent MS analysis were selected on the basis of the reproducibility of their identification as a DNA binding component as well as the gel resolution of proteins of similar size. The complexity in the protein expression profile, differential expression levels and the presence of co-migrating bands make it possible that not all proteins with a putative DNA binding activity would be detected. Thus, the procedure employed might yield only a selection of all DNA binding proteins co-purifying with the neisserial inner membrane (see Discussion).

### Identification of DNA binding proteins

DNA binding proteins excised from gels were trypsin-treated and identified by MS (Table 4). Among the inner membrane proteins identified that bound DNA were the competence protein ComL, the cell division ATP-binding protein FtsE, as well as two hypothetical proteins NMB0478 and NMB0086 (Fig. 2). Reports have suggested that the 29 kDa lipoprotein ComL (Fig. 2a[Fig f2]) contributes to cleavage of the peptidoglycan layer, providing access for incoming DNA to enter the bacterial cell. However, the main function of ComL is difficult to assess, since most mutants of this essential component are lethal (Fussenegger *et al.*, 1996). The cell division ATP-binding protein FtsE (Corbin *et al.*, 2007) was also identified as an inner membrane protein that binds DNA (Fig. 2). FtsE has been shown to be associated with the inner membrane in *E. coli* (Gill & Salmond, 1987), and is believed to be involved in cell division due to its interactions with cell division proteins FtsZ and FtsX. The sequences of FtsE and FtsX show homology to ABC transporters; FtsE makes up the ATP-binding cassette (ABC) component, while FtsX anchors the FtsEX complex to the membrane. FtsZ polymerizes into the Z ring and recruits the FtsEX complex to the division site (Corbin *et al.*, 2007; de Leeuw *et al.*, 1999; Schmidt *et al.*, 2004). Even though FtsE is essential in *E. coli*, studies of FtsE in *N. gonorrhoeae* indicate that the protein is non-essential in this organism (Bernatchez *et al.*, 2000). Furthermore, the two hypothetical proteins NMB0478 and NMB0086 identified are conserved among the neisserial species, but show no significant homology to other proteins of known function (Fig. 2a[Fig f2]).

In addition, the pilus-biogenesis protein PilG was found to exhibit DNA binding activity (Fig. 2). Again, no DUS specificity or preference for binding to single-stranded versus double-stranded DNA substrates was observed (Figs 2 and 3; and data not shown). PilG is a polytopic inner membrane protein; however, its exact function in pilus biogenesis is yet to be elucidated.

Among the DNA binding outer membrane proteins detected that co-purified with neisserial inner membranes were PilQ, H.8, Omp85 and opacity proteins, all of which are outer membrane proteins expressed at high levels in *Neisseria* species. Contamination of outer membrane proteins in the inner membrane fractions was assessed by monitoring the presence of the secretin PilQ (Collins *et al.*, 2001; Tønjum *et al.*, 1998). PilQ binds DNA without DUS specificity (Assalkhou *et al.*, 2007) and also directly interacts with the inner membrane lipoprotein PilP (Balasingham *et al.*, 2007), promoting its presence in inner membrane fractions. Omp85 is an outer membrane protein, highly conserved among Gram-negative bacteria. This protein has been shown to be essential for survival in *N. meningitidis* and plays a role in outer membrane assembly (Voulhoux *et al.*, 2003). H.8, also known as Lip in *N. meningitidis*, is a neisserial-specific outer membrane lipoprotein, primarily expressed in pathogenic *Neisseria* species and not so frequently expressed in commensal species (Cannon *et al.*, 1984; Hitchcock *et al.*, 1985; Hitchcock, 1989). One study has shown that the H.8 lipoprotein of *N. gonorrhoeae* stimulates the immune system, indicating a possible role in neisserial pathogenesis (Fisette *et al.*, 2003). Finally, opacity proteins are phase-variable outer membrane proteins that mediate adherence of pathogenic *Neisseria* spp. to host cells (Dehio *et al.*, 1998).

### Bioinformatics analysis of DNA binding components

The deduced amino acid sequences of the DNA binding proteins identified were screened for recognized DNA binding motifs such as helix–turn–helix, helix–hairpin–helix, helix–loop–helix, leucine zipper and zinc finger. No recognized DNA binding motifs were found in any of the candidate proteins. Furthermore, the genes encoding the two hypothetical proteins NMB0478 and NMB0086 contain DUS sequences, which might indicate that they have a role in DNA metabolism (Davidsen *et al.*, 2004; Treangen *et al.*, 2008).

### *N. meningitidis* mutant phenotypes

In order to assess the significance of the DNA binding activity of the proteins identified, the corresponding null mutants of candidates yielding highly reproducible results were constructed. The mutants were assessed with regard to colony morphology (which is dependent on the piliation state), expression of extracellular pili, and competence for transformation as compared with the wild-type strain (Table 4). Only the pilus biogenesis mutants Δ*pilG* and Δ*pilQ* were defective in competence, as well as in pilus expression, and showed a non-agglutinating colony morphology (Table 4).

## DISCUSSION

The full complement of DNA binding proteins involved in neisserial transformation is yet to be unravelled. The outer membrane complex PilQ has previously been shown to bind DNA. In order to define inner membrane proteins engaged in transformation, we searched for DNA binding components in inner membrane fractions of *N. meningitidis* by a solid-phase overlay assay and tested the corresponding null mutants with regard to competence for transformation. A number of proteins exhibited DNA binding activity in this *in vitro* assay, and the predominant eight of these were identified by MS. However, only pilus biogenesis components exerted an effect on transformation. The finding that pilus biogenesis proteins directly bind DNA further supports the already established link between pilus biogenesis and uptake of DNA by natural transformation in *N. meningitidis*.

Searching for DNA binding proteins by a solid-phase overlay assay presents both opportunities and limitations. The main advantage of the method is that by coupling it to SDS-PAGE, it is possible to separate complex mixtures of proteins, assess the DNA binding abilities of proteins separately and identify DNA binding proteins by MS. During the SDS-PAGE step, proteins are, however, denatured, and if their DNA binding activity is dependent on correct folding, the proteins have to be able to renature under the conditions employed in order to be detected. In addition, the proteins have to be expressed in high enough amounts to give distinct and detectable bands in Coomassie blue-stained gels, enabling excision and identification of proteins by MS. However, some proteins might not renature under the conditions employed and/or are expressed at too low levels to yield a distinct band in SDS-PAGE. In addition, the accuracy of protein excision might vary due to co-migration in the PAGE system. Thus, only a predominant and selected subset of all potential DNA binding proteins in a sample will be detected by this method.

As for any method measuring *in vitro* protein–DNA interactions, the detection of an interaction in the current assay would only be indicative with respect to the relevance to the biological system under study. Furthermore, due to positive electrostatic charge, DNA binding activity detected *in vitro* might be non-specific; however, recognized DNA binding motifs also exhibit positive electrostatic charge. Despite these limitations, the strategy employed is a useful approach for screening complex mixtures of proteins and identifying candidates with DNA binding activity. These represent a basis for further testing. In this context, we focused on proteins involved in transformation, which is the major source of the abundance of exogenous DNA introduced into the meningococcal chromosome.

Null mutants corresponding to the DNA binding proteins identified in neisserial inner membranes were constructed and tested for competence for transformation. Only the PilG and PilQ mutants, defective in pilus biogenesis, were non-competent for transformation. For these two components, the interpretation of the biological significance of their DNA binding capabilities is complicated by the fact that they participate in type IV pilus biogenesis, which is required for competence. Thus, it is a conundrum whether the lack of competence in these mutants is due to a defect in their direct binding of DNA, or whether it is indirect, through pilus biogenesis. The DNA binding activity of PilQ in a solid-phase overlay assay has previously been documented, and the PilQ–DNA interaction has also been verified by electromobility shift analysis (EMSA) and mapped to the pore region of the PilQ complex by electron microscopy (Assalkhou *et al.*, 2007). Those findings represent validation of the solid-phase overlay approach for the identification of DNA binding proteins in general. Nevertheless, the biological significance and nature of PilQ DNA binding activity remains to be fully elucidated. The DNA binding activity of PilG has, however, not been previously described.

The null mutants for the cell division ATP-binding protein FtsE, the outer membrane protein H.8 and the two hypothetical proteins NMB0478 and NMB0086 were all competent for transformation. Interestingly, the meningococcal Δ*ftsE* mutant was viable. Even if no effect on transformation was observed, a potential role for their observed DNA binding activity in other cellular events such as conjugation, replication and other DNA metabolism cannot be excluded. However, defining the context of their DNA binding activity was beyond the scope of this study.

For two of the DNA binding proteins identified, ComL and Omp85, viable mutants could not be made, suggesting that they are essential. We were therefore unable to test whether their observed DNA binding activity contributes to transformation. However, ComL has been suggested to play a role in the peptidoglycan-related phase of transformation (Fussenegger *et al.*, 1996). Thus, further studies are needed to determine whether the observed DNA binding has any biological significance in transformation. Whether the DNA binding noted for the proteins Omp85 and H.8 is significant or not must also be assessed in light of the relative abundance of these proteins in the outer membrane.

The ability to take up exogenous DNA is of great importance when it comes to bacterial fitness, survival and evolution (Ochman *et al.*, 2000). The uptake of DNA in *N. meningitidis* is dependent on DUS sequences, which are present in large numbers throughout the neisserial genome. Interestingly, it has been shown that DUS sequences inside ORFs are preferentially located in genome maintenance and repair genes (Davidsen *et al.*, 2004), indicating that transforming DNA has a function in DNA repair (Treangen *et al.*, 2008). The preferential uptake of DNA containing DUS sequences makes the bacterium more prone to take up DNA from itself and/or closely related species. One central question is how *N. meningitidis* manages to bind, take up and incorporate new DNA into the genome. The discovery of a DUS-specific receptor would be a great milestone in the elucidation of neisserial natural transformation. A DNA binding protein that exerts DUS specificity in DNA binding was not detected in this study of inner membrane components. It is still an open question whether the discrimination between DUS-positive and DUS-negative DNA occurs before or after the transforming DNA reaches the inner membrane. Since the secretin PilQ appears to function as a non-selective gateway for DNA through the outer membrane, DUS specificity might be exerted in the periplasm/peptidoglycan layer, the inner membrane, or during recombination. In this context, the continued search for neisserial DNA binding components is highly relevant.

## Figures and Tables

**Fig. 3. f3:**
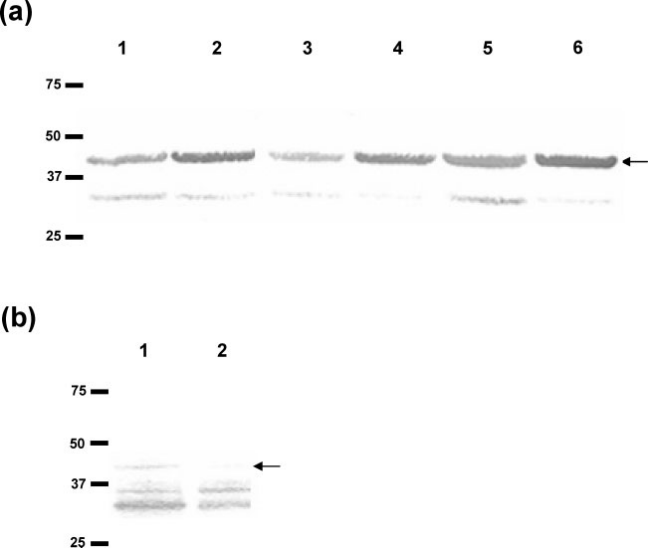
PilG binds DNA without DUS specificity. Solid-phase overlay assay (South-western analysis) shows DNA binding activity of recombinant PilG protein and the PilG protein from the meningococcal inner membrane fraction. No DUS specificity was observed and the position of the PilG protein is indicated by the arrow. (a) DNA binding activity of full-length recombinant PilG. Lanes: 1, ssDNA substrate (T_1_) with 10 bp DUS; 2, dsDNA substrate (T_1_T_2_) with 10 bp DUS; 3, ssDNA substrate (T_3_) without DUS; 4, dsDNA substrate (T_3_T_4_) without DUS; 5, ssDNA substrate (HH8) with 12 bp DUS; 6, dsDNA substrate (HH7,HH8) with 12 bp DUS. (b) Identification of PilG DNA binding activity in the meningococcal inner membrane fraction using ssDNA substrate (T_1_). Lane 1, H44/76 wild-type; lane 2, H44/76 ΔPilG. The positions of the size standards (kDa) are shown on the left.

**Table 1. t1:** Bacterial strains and plasmids employed in this study

**Strain or plasmid**	**Relevant characteristics**	**Reference or source**
***N. meningitidis* strains**		
H44/76 wild-type	Serogroup B, isolated in Norway, 1976	Holten (1979)
H44/76 ΔPilG	*pilG* : : mTnErm transposon insertion	Tønjum *et al.* (1995)
MC58 wild-type	Serogroup B, isolated in the UK, 1983	McGuinness *et al.* (1991)
Z2491 wild-type	Serogroup A, isolated in Gambia, 1983	Achtman *et al.* (1991); Crowe *et al.* (1989)
8013 wild-type	Serogroup C	Caugant *et al.* (1986)
M1080 wild-type	Serogroup B, isolated in the USA, 1969	Frasch & Chapman (1972)
M1080 ΔPilQ	M1080 strain with mTnCm#21 in *pilQ*	Tønjum *et al.* (1998)
M400 (M1080-A)	Derivative of M1080 with *recA6* (*tetM*)*	Tønjum *et al.* (1998)
***N. gonorrhoeae* strain**		
N400 wild-type	*recA6* (*tetM*)	Tønjum *et al.* (1995)
***E. coli* strains**		
ER2566	Expression strain with a chromosomal copy of the T7 RNA polymerase gene	New England Biolabs
XL-1 Blue	*recA1 endA1 gyrA96 thi-1 hsdR17 supE44 relA1 lac* [F′ *proAB lacI^q^Z*Δ*M15* Tn*10*Tet^r^]	Stratagene
**Vectors**		
p1080*mutY-erm*^r^	Plasmid containing an erythromycin-resistance gene inserted into *mutY*	Davidsen *et al.* (2007b)
pBSK+	General cloning vector, Amp^r^	Stratagene

*recA6 is an IPTG-inducible allele of *recA*.

**Table 2. t2:** DNA substrates employed in the study DUS sequences are underlined.

**Substrate**	**Sequence**	**DUS length**
**ssDNA**		
T_1_	5′ CAACAACAACAACAGCCGTCTGAACCAAATTCAGACGGCAACAACAACAACA 3′	+, 10 bp
T_3_	5′ CAACAACAACAACAGGCCTGTCATCCAAACTGACAGGCCAACAACAACAACA 3′	−
HH8	5′ GTTGTTGTTGTTTTCAGACGGCATGTTGGTTCAGACGGCATTTGTTGTTGTT 3′	+, 12 bp
**dsDNA**		
T_1_T_2_	5′ CAACAACAACAACAGCCGTCTGAACCAAATTCAGACGGCAACAACAACAACA 3′	+, 10 bp
	5′ TGTTGTTGTTGTTGCCGTCTGAATTTGGTTCAGACGGCTGTTGTTGTTGTTG 3′	
T_3_T_4_	5′ CAACAACAACAACAGGCCTGTCATCCAAACTGACAGGCCAACAACAACAACA 3′	−
	5′ TGTTGTTGTTGTTGGCCTGTCATTTTGGATGACAGGCCTGTTGTTGTTGTTG 3′	
HH7,HH8	5′ AACAACAACAAATGCCGTCTGAACCAACATGCCGTCTGAAAACAACAACAAC 3′	+, 12 bp
	5′ GTTGTTGTTGTTTTCAGACGGCATGTTGGTTCAGACGGCATTTGTTGTTGTT 3′	

**Table 3. t3:** Primers employed in construction of mutants Restriction sites are underlined.

**Primer**	**Orientation**	**Sequence (5′→3′)**	**Restriction site**
**FtsE (NMB0007)**			
KH83	Forward	GCGCTAGCGTTTCGAACAAGTTTCCAAA	*Nhe*I
KH10	Reverse	GCGGATCCCATCAGGGTTTCGTCATGTG	*Bam*HI
KH11	Reverse	GCGAATTCGGATCATAGGAGGTCCTGTAA	*Eco*RI
KH12	Forward	GCAAGCTTCAAATGGTACAAAGCGCAGA	*Hin*dIII
**ComL (NMB0703)**			
KH45	Forward	GCGCTAGCTCTTGGGTAATCTGGGCATC	*Nhe*I
KH46	Reverse	GCGGATCCGTTTGTTGACGACGATGACG	*Bam*HI
KH47	Reverse	GCGAATTCACGAGCTGAACAGCAGCAAT	*Eco*RI
KH48	Forward	GCAAGCTTCAATATGGCGAGCGATTCTT	*Hin*dIII
**Hypothetical protein (NMB0478)**			
KH82	Forward	GCGCTAGCGAATCAACACTTTCACTACAAGCAA	*Nhe*I
KH6	Reverse	GCGGATCCTTCCATCTGTGCGACTTCTG	*Bam*HI
KH7	Reverse	GCAAGCTTTGAAAGTGTTGATTCCATATTAAAC	*Hin*dIII
KH8	Forward	GCGGTACCCTTCCGCATCCGATATGACT	*Kpn*I
**Hypothetical protein (NMB0086)**			
KH13	Forward	GCGAATTCCTCATTGCGCTGCCGTTT	*Eco*RI
KH14	Reverse	GCGGATCCCTTGCCGTCTATCATCACGA	*Bam*HI
KH15	Reverse	GCGAATTCCGATACATGATGTTCCTTCCAAA	*Eco*RI
KH16	Forward	GCAAGCTTCCAAATTCGTGTGGTGTCTG	*Hin*dIII
**H.8 (NMB1533)**			
KH81	Reverse	GCGCTAGCGTATCTGGCTCTGATTTCTG	*Nhe*I
KH2	Forward	GCAAGCTTCTTCGCCGCCGCCGATCAGTT	*Hin*dIII
KH3	Forward	GCGAATTCGCTTTCATAACAAATCTCCAAT	*Eco*RI
KH4	Reverse	GCGGATCCCATTTTCAGACGGCATGTAT	*Bam*HI
**Omp85 (NMB0182)**			
KH84	Reverse	GCGCTAGCGCATATCGCCTTTGGCACTT	*Nhe*I
KH18	Forward	GCAAGCTTTTCAAATTCGATGTCGGTGA	*Hin*dIII
KH19	Forward	GCGAATTCAGTTCCTTGTGGTGCGGAA	*Eco*RI
KH20	Reverse	GCGGATCCAGCTACCGTCCGTCTGTTGT	*Bam*HI

**Table 4. t4:** Phenotypic traits of null mutants corresponding to the DNA binding proteins identified by solid-phase overlay assay and MS analysis

Protein identified*	**Putative function**	**Colony morphology†**	**Extracellular pilus expression**	**Competence for transformation**
NMB0007 FtsE DUS−	ATPase activity, cell division	agg+	wt‡	Yes
NMB0703 ComL DUS−	Competence, peptidoglycan function	Not viable	Not viable	Not viable
NMB0478 hypothetical protein DUS+	Unknown	agg+	wt	Yes
NMB0086 hypothetical protein DUS+	Unknown	agg+	wt	Yes
NMB0333 PilG DUS−	Pilus biogenesis?	agg−	Absent	Non-competent (Tønjum *et al.*, 1995)
NMB1812 PilQ DUS−	Outer membrane pore, pilus extrusion transformation	agg−	Absent	Non-competent (Frye *et al.*, 2006)
NMB1533 H.8 DUS−	Iron–sulfur binding?	agg+, smaller than wt	wt	Yes
NMB0182 Omp85 DUS−	Cell envelope biogenesis	Not viable	Not viable	Not viable

*The presence/absence of the DUS is given as DUS+/DUS−. †Colony morphology is described as agglutinating (agg+) or non-agglutinating (agg−). ‡wt, Wild-type levels.

## Abbreviations

- DUS, DNA uptake sequence
- OMV, outer membrane vesicle
